# A Comparative Study of the Visual Outcomes of Two Common Diffractive Trifocal Intraocular Lenses in a Matched Cohort

**DOI:** 10.7759/cureus.114447

**Published:** 2026-08-12

**Authors:** Zahraa O Alomar, Hanna Pawlowski, Sanjana Molleti, Noor Virk, Zahra Adamji, Mina M Sitto, Parsa Khaksar, Triston B Crook, Majid Moshirfar

**Affiliations:** 1 Department of Ophthalmology, University of Washington, Seattle, USA; 2 Hoopes Moshirfar Research Center, Hoopes Vision, Draper, USA; 3 Department of Ophthalmology and Visual Neurosciences, University of Minnesota, Minneapolis, USA; 4 Department of Biological Sciences, Arizona State University, Tempe, USA; 5 Department of Ophthalmology, University of Arizona College of Medicine - Phoenix, Phoenix, USA; 6 John A. Moran Eye Center, University of Utah, Salt Lake City, USA; 7 Department of Ophthalmology, Louisiana State University Health Sciences Center Shreveport, Shreveport, USA; 8 School of Medicine, Noorda College of Osteopathic Medicine, Provo, USA; 9 Department of Eye Banking and Corneal Transplantation, Utah Lions Eye Bank, Murray, USA

**Keywords:** intraocular lens, iols, odyssey, ophthalmology, outcomes, panoptix pro, trifocal

## Abstract

Purpose

The purpose of this study was to compare monocular and binocular visual acuity, refractive outcomes, and visual symptom outcomes at one month postoperatively between two recently released trifocal intraocular lens (IOL) models: the TECNIS Odyssey (Johnson & Johnson Vision, Santa Ana, CA, USA) and the Clareon PanOptix Pro (Alcon Vision, Fort Worth, TX, USA).

Methods

A retrospective analysis was conducted of patients who underwent cataract extraction with implantation of either the TECNIS Odyssey or Clareon PanOptix Pro IOL. The groups were matched 1:1 using nearest-neighbor matching based on baseline corneal astigmatism to correct for baseline imbalance. Generalized estimating equations were used to account for inter-eye correlation. Primary outcomes included monocular and binocular uncorrected distance visual acuity (UDVA), uncorrected near visual acuity (UNVA), corrected distance visual acuity (CDVA), and manifest refraction. Patient-reported photic symptoms (glare, halos, photophobia, and night vision difficulty) were evaluated as secondary outcomes.

Results

A total of 168 patients (246 eyes) were initially included: 50 patients (66 eyes) in the Odyssey IOL group and 118 patients (180 eyes) in the PanOptix Pro IOL group. After matching, a total of 130 eyes (65 eyes per IOL group) were analyzed. Mean monocular UDVA was not significantly different between the Odyssey and PanOptix Pro groups (logMAR 0.10 ± 0.11 vs. 0.13 ± 0.13; p = 0.29), although Odyssey eyes were more likely to achieve the 20/50 or better threshold (56 eyes, 100% vs. 49 eyes, 89.1%; p = 0.013). The Odyssey group demonstrated a small but statistically significant advantage in CDVA (logMAR -0.001 ± 0.097 vs. 0.039 ± 0.08; p = 0.034). However, UNVA, binocular visual acuity, and manifest refraction did not differ significantly between the IOL groups. Patient-reported visual symptoms remained below a 15% incidence and were statistically comparable between groups.

Conclusions

TECNIS Odyssey and Clareon PanOptix Pro demonstrated generally comparable early visual, refractive, and photic outcomes in this retrospective matched cohort of uncomplicated eyes. A small but statistically significant difference in CDVA favored Odyssey, although its clinical relevance remains uncertain.

## Introduction

Approximately 94 million people worldwide experience visual impairment due to cataracts [1]. Cataract surgery has become the mainstay of treatment, with rapid advancements in intraocular lens (IOL) technology [2]. Monofocal lenses provide correction at a single focal point; however, patients remain dependent on eyeglasses [3]. Trifocal lenses address this limitation by splitting incoming light across three focal points to provide distance, intermediate, and near vision, and meta-analyses have shown that they outperform extended-depth-of-focus (EDOF) designs for near tasks [4,5]. However, diffractive light-splitting reduces contrast sensitivity and can produce halos, glare, and starbursts, most noticeably at night [6,7].

Two recently introduced trifocal platforms have aimed to reduce the dependence on eyeglasses associated with monofocal lenses. Both lenses were introduced only recently, with the Clareon PanOptix Pro (Alcon Vision, Fort Worth, TX, USA) released in May 2025 and the TECNIS Odyssey (Johnson & Johnson Vision, Santa Ana, CA, USA) released in October 2024. Direct comparative data between these two lenses remain sparse [8].

The Clareon PanOptix Pro is the newest version of the PanOptix trifocal, built on the Clareon hydrophobic acrylic material and featuring a refined diffractive design marketed as ENhanced LIGHT ENergy (ENLIGHTEN NXT). Before the release of the PanOptix Pro, Alcon introduced the Clareon PanOptix lens (Alcon Vision), which addressed the glistenings observed in the earlier AcrySof IQ PanOptix lens (Alcon Vision). Glistenings are small, fluid-filled vacuoles within the IOL that develop specifically in hydrophobic acrylic material due to phase separation induced by changes in the surrounding temperature [9,10]. According to the manufacturer’s technical specifications, the TECNIS Odyssey was developed as an improved design based on Johnson & Johnson’s previous trifocal platform, the TECNIS Synergy IOL (Johnson & Johnson Vision). By using a glistening-free material, the IOL was designed to provide greater tolerance to refractive errors and an improved photic profile [8,11]. The TECNIS Odyssey combines multifocal and EDOF optics in a continuous freeform diffractive profile and incorporates chromatic aberration correction intended to preserve contrast and enhance an extended range of vision [11].

The purpose of this study was to compare distance and near visual outcomes between eyes implanted with the TECNIS Odyssey and the Clareon PanOptix Pro. Monocular uncorrected distance visual acuity (UDVA) and uncorrected near visual acuity (UNVA) at one month served as the primary endpoints, whereas binocular visual outcomes, refractive outcomes, and visual symptoms were evaluated as secondary endpoints.

## Materials and methods

This nonrandomized, retrospective, single-center study compared patients who underwent cataract surgery or refractive lens exchange with implantation of either the TECNIS Odyssey (between October 2024 and June 2026) or the Clareon PanOptix Pro (between October 2025 and June 2026), performed by four surgeons at Hoopes Vision, Draper, UT, USA. Each patient received a single IOL design; therefore, the two lenses were compared between independent cohorts. The study protocol was approved by the Hoopes Vision Ethics Committee and the Institutional Review Board of the Biomedical Research Alliance of New York (A20-12-547-823). This study was conducted in accordance with the Declaration of Helsinki, and all patients provided written informed consent before enrollment.

For inclusion, patients were required to have undergone phacoemulsification with a target of emmetropia, received identical IOL models in both eyes if bilateral surgery was performed, and completed at least one month of postoperative follow-up. Eligibility was further assessed on a per-eye basis: an eye qualified if both UDVA and UNVA were recorded at the one-month postoperative visit. In patients who underwent unilateral implantation, the operative eye served as the study eye if it met the eligibility criteria. In patients who underwent bilateral implantation, both eyes were included if the same IOL model had been implanted in each eye.

Eyes were excluded if they underwent monovision targeting; had a history of previous ocular surgery, significant ocular trauma, irregular corneal astigmatism, abnormal corneal topography, or ocular comorbidities affecting vision, such as glaucoma, diabetic retinopathy, or Fuchs’ dystrophy; or experienced any intraoperative or postoperative complication that could independently affect the visual outcome (e.g., capsular tear, IOL decentration, cystoid macular edema, or retinal detachment).

Patients underwent a comprehensive ocular examination using slit-lamp biomicroscopy. Refraction and visual acuity were recorded preoperatively and at postoperative day 1, week 1, month 1, and month 3, when available. Outcomes of interest included monocular UDVA, monocular UNVA, CDVA, the spherical and cylindrical components of manifest refraction, and spherical equivalent (SEQ). Corrected and uncorrected visual acuities were measured by an optometrist using a Snellen eye chart (M&S Technologies Inc., Niles, IL, USA). Distance visual acuity was assessed using a high-resolution display at 4 m. Near visual acuity was assessed at 40 cm. All near vision measurements were converted from Jaeger scores to Snellen visual acuity and then to the logarithm of the minimum angle of resolution (logMAR) using the following standard formula for statistical analysis:

$\mathrm{logMAR}=\log_{10}\left(\frac{1}{\text{decimal Snellen fraction}}\right)$

Secondary postoperative outcomes included patient-reported visual symptoms, including glare, halos, photophobia, and night vision difficulties. Symptoms were documented as binary outcomes (present vs. absent) based on routine clinical assessment at the postoperative visit rather than a standardized questionnaire.

Surgery was performed by four surgeons using standard small-incision phacoemulsification under topical anesthesia with in-the-bag IOL implantation. A manual 2.25-mm clear corneal incision was created with a keratome, followed by a 5.0-5.5-mm continuous curvilinear capsulorhexis. Phacoemulsification was performed using either the horizontal chop or divide-and-conquer technique with the Infiniti Vision System (Alcon Laboratories, Inc., Fort Worth, TX, USA).

The two study lenses were the TECNIS Odyssey and the Clareon PanOptix Pro. Odyssey eyes received either the non-toric model (DRN00V) or toric models (DRT150 or DRT225). PanOptix Pro eyes received either the non-toric model (PXCAT0) or toric models (PXCAT3-PXCAT6). Because the toric and non-toric versions of each lens share the same diffractive optic, all eyes were pooled within their respective groups for the visual acuity analyses.

Preoperative data extracted from the medical records included manifest refraction, intraocular pressure (IOP), slit-lamp examination findings, and dilated fundus examination findings. Optical biometry was performed using either the IOLMaster 700 (Carl Zeiss Meditec, Jena, Germany) or the LenStar LS 900 (Haag-Streit, Köniz, Switzerland), and the device used was recorded for each eye. Collected biometric parameters included keratometry (flat (K1) and steep (K2) meridian powers and their corresponding axes), mean keratometry, axial length, anterior chamber depth, aqueous depth, lens thickness, central corneal thickness (CCT), and white-to-white diameter. Corneal apex pachymetry was obtained using Scheimpflug tomography (Pentacam, Oculus, Wetzlar, Germany) or a Galilei dual-Scheimpflug analyzer when available. IOL power, cylinder, and axis were recorded for every eye.

A post hoc power analysis for the difference between two independent means was performed using G*Power (version 3.1.9.7). Assuming an effect size (d) of 0.5, an alpha level of 0.05, and a sample size of 65 eyes per group (130 eyes in total), the resulting statistical power was 0.81. However, this estimate does not account for the inter-eye correlation addressed in the generalized estimating equation (GEE) models; therefore, the true power to detect a between-group difference is likely lower. Using the patient-level sample size (n = 49), the statistical power was 0.69, suggesting that the true power to detect a moderate effect size likely falls between these two estimates.

Statistical analyses were performed using Python (version 3.14; Python Software Foundation, Wilmington, DE, USA) and Microsoft Excel (version 16.110.3; Microsoft Corporation, Redmond, WA, USA). Continuous variables are presented as the mean ± SD, and categorical variables as counts and percentages. GEE models with an exchangeable working correlation structure were used to account for inter-eye correlation. When visual acuity success rates approached 100%, Fisher’s exact test was used to assess statistical significance. Symptom frequencies were compared using the χ² test or Fisher’s exact test. At the patient level, Welch’s t-test was used to compare age, and the chi-square test was used to compare sex. Statistical significance was defined as p < 0.05. Because baseline corneal astigmatism (ΔK) differed significantly between groups, matching was performed based on baseline ΔK. One-to-one nearest-neighbor matching without replacement, using a caliper of 0.2, was performed with the MatchIt package in RStudio (version 4.6.1; Posit Software, PBC, Boston, MA, USA).

Throughout this study, the TECNIS Odyssey and Clareon PanOptix Pro IOLs are hereafter referred to as Odyssey and PanOptix Pro, respectively.

## Results

Demographics and baseline characteristics

A total of 168 patients (246 eyes) were initially included: 50 patients (66 eyes) in the Odyssey IOL group and 118 patients (180 eyes) in the PanOptix Pro IOL group. However, the Odyssey IOL group had significantly greater mean corneal astigmatism (ΔK: 0.79 ± 0.51 D vs. 0.54 ± 0.61 D; p < 0.001). Simple covariate matching was performed to account for this difference, and the matched baseline characteristics are presented in Table 1. The matching process resulted in a total of 98 patients (130 eyes), with 49 patients (65 eyes) in each IOL group. There were no significant differences in age, sex, preoperative SEQ, IOL power, or biometric parameters between the groups. However, the standardized mean differences (SMDs) for IOP and CCT remained above the conventional threshold of <0.25, suggesting that some imbalance remained after matching.

**Table 1:** Preoperative characteristics of the study groups. ^†^ Welch’s two-sample t test ^‡^ Pearson’s χ² test ^¶^ GEE with a Gaussian distribution, an exchangeable correlation structure, and the Wald test to account for inter-eye correlation. The study groups were matched for corneal astigmatism, calculated as the difference between the mean steep (K2) and mean flat (K1) keratometry values. SEQ was calculated as the spherical component plus one-half of the cylindrical component of the manifest refraction. ACD, anterior chamber depth; AL, axial length; CCT, central corneal thickness; GEE, generalized estimating equation; IOL, intraocular lens; IOP, intraocular pressure; LT, lens thickness; SEQ, spherical equivalent; SMD, standardized mean difference

Preoperative parameter	Odyssey	PanOptix Pro	p-value	SMD	Test statistic
Patient characteristics					
Patients, n	49	49	-	-	-
Eyes, n	65	65	-	-	-
Sex (male/female), n (%)	25 (51.0)/24 (49.0)	23 (46.9)/26 (53.1)	0.6082	0.082	0.28^‡^
Age (years), mean ± SD	64.31 ± 10.99	65.37 ± 9.37	0.5969	0.104	-0.51^†^
Eye characteristics					
Sphere (D), mean ± SD	-0.44 ± 2.99	0.07 ± 1.95	0.1703	0.202	1.37^¶^
Cylinder (D), mean ± SD	-0.92 ± 0.61	-0.96 ± 0.70	0.8688	0.057	0.17^¶^
SEQ (D), mean ± SD	-0.90 ± 3.04	-0.40 ± 1.96	0.1651	0.195	1.39^¶^
ΔK (D), mean ± SD	0.80 ± 0.51	0.81 ± 0.50	0.982	0.005	-0.02^¶^
IOP (mmHg), mean ± SD	14.06 ± 2.95	12.98 ± 3.57	0.1068	0.33	-1.61^¶^
AL (mm), mean ± SD	23.93 ± 1.49	23.81 ± 1.10	0.53	0.095	-0.63^¶^
ACD (mm), mean ± SD	3.24 ± 0.33	3.25 ± 0.39	0.8627	0.033	0.03^¶^
CCT (μm), mean ± SD	537.95 ± 30.01	550.52 ± 33.78	0.2787	0.393	1.08^¶^
LT (mm), mean ± SD	4.40 ± 0.45	4.45 ± 0.33	0.3506	0.141	0.93^¶^
Toric/non-toric, n (%)	12 (18.5) / 53 (81.5)	20 (30.8) / 45 (69.2)	0.354	0.132	2.27^‡^
IOL power (D), mean ± SD	19.57 ± 4.67	20.03 ± 2.92	0.5478	0.119	0.60^¶^

Visual acuity outcomes

**Figure 1:**
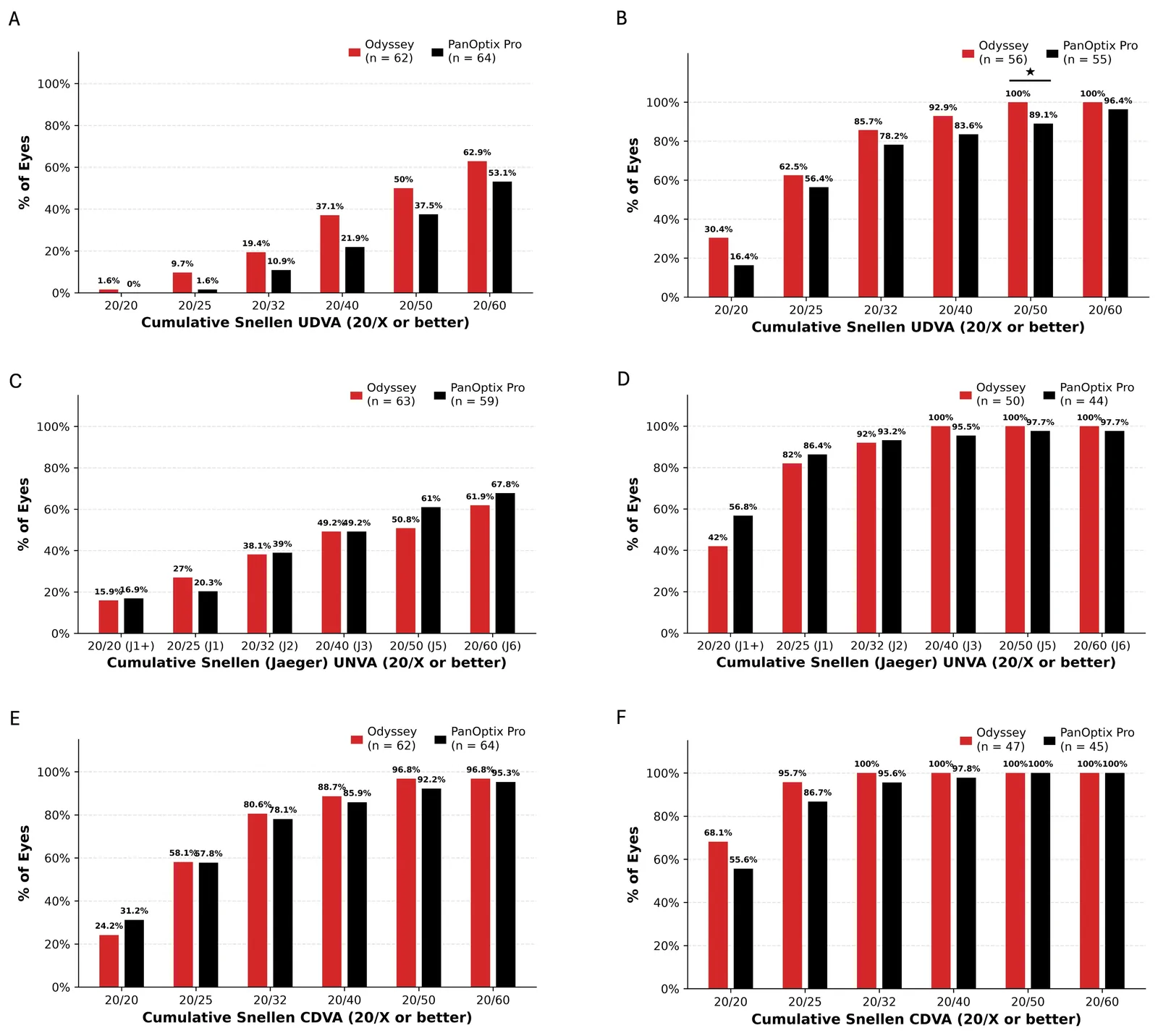
Percentage of eyes achieving a cumulative Snellen 20/X or better. Percentage of eyes achieving a cumulative Snellen 20/X or better for (A) preoperative monocular UDVA, (B) one-month postoperative monocular UDVA, (C) preoperative monocular UNVA, (D) one-month postoperative monocular UNVA, (E) preoperative CDVA, and (F) one-month postoperative CDVA for the Odyssey (TECNIS Odyssey) and PanOptix Pro (Clareon PanOptix Pro) IOLs. An asterisk (*) indicates a statistically significant difference (p < 0.05). Due to the retrospective nature of the study and its limited one-month evaluation period, complete visual acuity data were not available for all included eyes. Statistical test: Logistic GEE models with a binomial distribution and an exchangeable correlation structure were used to account for inter-eye correlation. CDVA, corrected distance visual acuity; GEE, generalized estimating equation; IOL, intraocular lens; UDVA, uncorrected distance visual acuity; UNVA, uncorrected near visual acuity

Of the 130 matched eyes, monocular UDVA data were available for 56 Odyssey eyes and 55 PanOptix Pro eyes; monocular UNVA data were available for 50 and 44 eyes, respectively; and CDVA data were available for 47 and 45 eyes, respectively. Preoperatively, the distribution of UDVA outcomes was similar between groups across all thresholds (Figure 1A).

When evaluating the distribution of UDVA outcomes, Odyssey demonstrated a significant advantage at the 20/50 threshold, with 100% of eyes achieving this level compared with only 89.1% of PanOptix Pro eyes (p = 0.013; Figure 1B). However, this threshold-level comparison did not correspond to a significant difference in overall mean UDVA between groups (p = 0.29). Postoperative UDVA was not statistically significantly different between the two groups (Odyssey: 0.10 ± 0.11; PanOptix Pro: 0.13 ± 0.13; p = 0.29).

When evaluating the distribution of near vision outcomes, Odyssey and PanOptix Pro demonstrated similar outcomes across all thresholds (Figure 1C, 1D). At the reading threshold (J1, 20/25), 82% and 86.4% of Odyssey and PanOptix Pro eyes, respectively, achieved this threshold one month postoperatively. Similarly, 42% of Odyssey and 56.8% of PanOptix Pro eyes achieved the J1+ (20/20) threshold at the one-month postoperative visit (p = 0.139).

**Figure 2:**
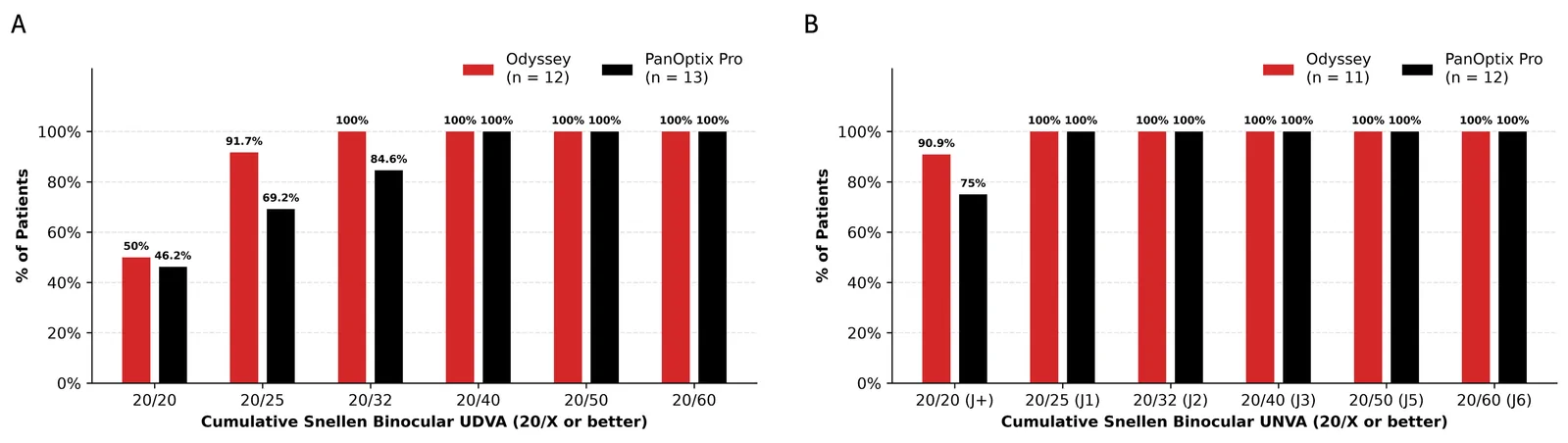
Percentage of patients achieving a cumulative Snellen 20/X or better. (A) One-month postoperative binocular UDVA and (B) one-month postoperative binocular UNVA for the Odyssey (TECNIS Odyssey) and PanOptix Pro (Clareon PanOptix Pro) IOLs. Due to the retrospective nature of the study and its limited one-month evaluation period, complete visual acuity data were not available for all included patients. IOL, intraocular lens; J, Jaeger; UDVA, uncorrected distance visual acuity; UNVA, uncorrected near visual acuity

Preoperatively, the distribution of CDVA outcomes was similar between groups across all thresholds (Figure 1E). For corrected visual acuity, there were no significant differences across the thresholds (Figure 1F); however, Odyssey demonstrated a slight but statistically significant advantage over PanOptix Pro (mean logMAR of -0.001 ± 0.097 vs 0.039 ± 0.08; p = 0.034). There were also no statistically significant differences between the Odyssey and PanOptix Pro IOL groups in binocular visual acuity testing for distance (0.015 ± 0.051 logMAR vs 0.077 ± 0.091 logMAR; p = 0.22) or near (-0.036 ± 0.067 logMAR vs -0.038 ± 0.069 logMAR; p = 0.92), or across the thresholds (Figure 2A, 2B).

Refractive outcomes

**Figure 3:**
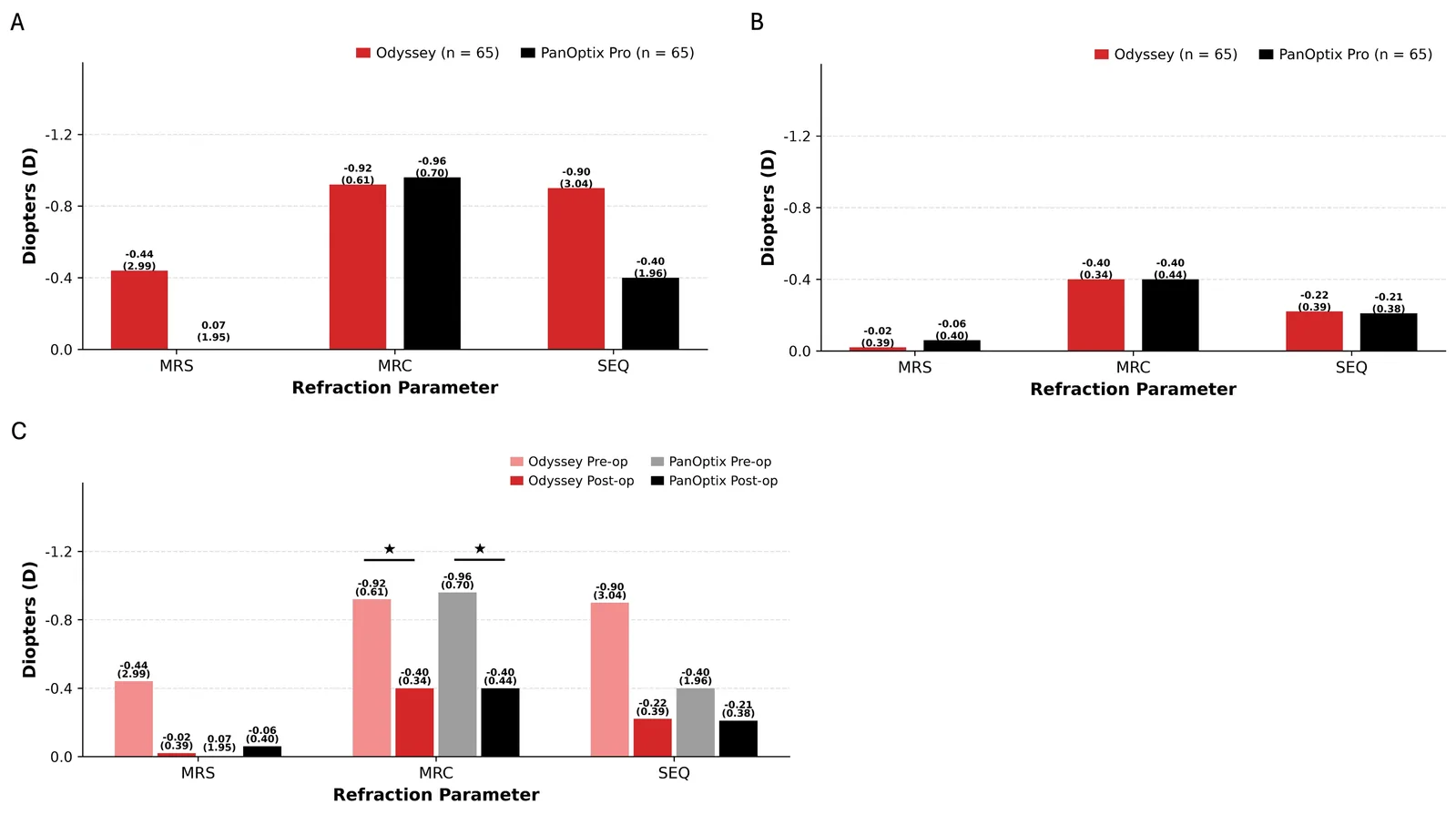
Mean manifest refraction (sphere, cylinder, and SEQ) measurements. Mean manifest refraction (sphere, cylinder, and SEQ) measurements (A) preoperatively, (B) at one month postoperatively, and (C) preoperative and one-month postoperative measurements for the Odyssey (TECNIS Odyssey) and PanOptix Pro (Clareon PanOptix Pro) IOLs. An asterisk (*) indicates a statistically significant difference (p < 0.05). Mean values are presented outside the parentheses, with the corresponding SDs shown within the parentheses. SEQ was calculated as the spherical component plus one-half of the cylindrical component of the manifest refraction. Statistical test: Gaussian GEE models with an exchangeable correlation structure were used to account for inter-eye correlation. GEE, generalized estimating equation; IOL, intraocular lens; MRC, cylindrical component of manifest refraction in diopter cylinder; MRS, spherical component of manifest refraction in diopters; SEQ, spherical equivalent

Preoperative (Figure 3A) and one-month postoperative (Figure 3B) refractive outcomes were comparable between the groups across all three parameters (sphere, cylinder, and SEQ). At one month postoperatively, both lenses achieved comparable mean refractive sphere (Odyssey: -0.02 ± 0.39 D vs. PanOptix Pro: -0.06 ± 0.40 D; p = 0.66), mean refractive cylinder (-0.40 ± 0.34 D vs. -0.40 ± 0.44 D; p = 0.99), and SEQ (-0.22 ± 0.39 D vs. -0.21 ± 0.38 D; p = 0.75; Figure 3B). Both lenses demonstrated a reduction in mean refractive sphere following surgery, with no significant between-group differences. The percentages of eyes with a manifest refraction spherical equivalent ≤0.50 D were 81.1% in the Odyssey group and 86.2% in the PanOptix Pro group (p = 0.6155). Complete statistical analyses of the visual acuity and refractive outcomes are provided in Appendix A.

Visual symptoms

**Figure 4:**
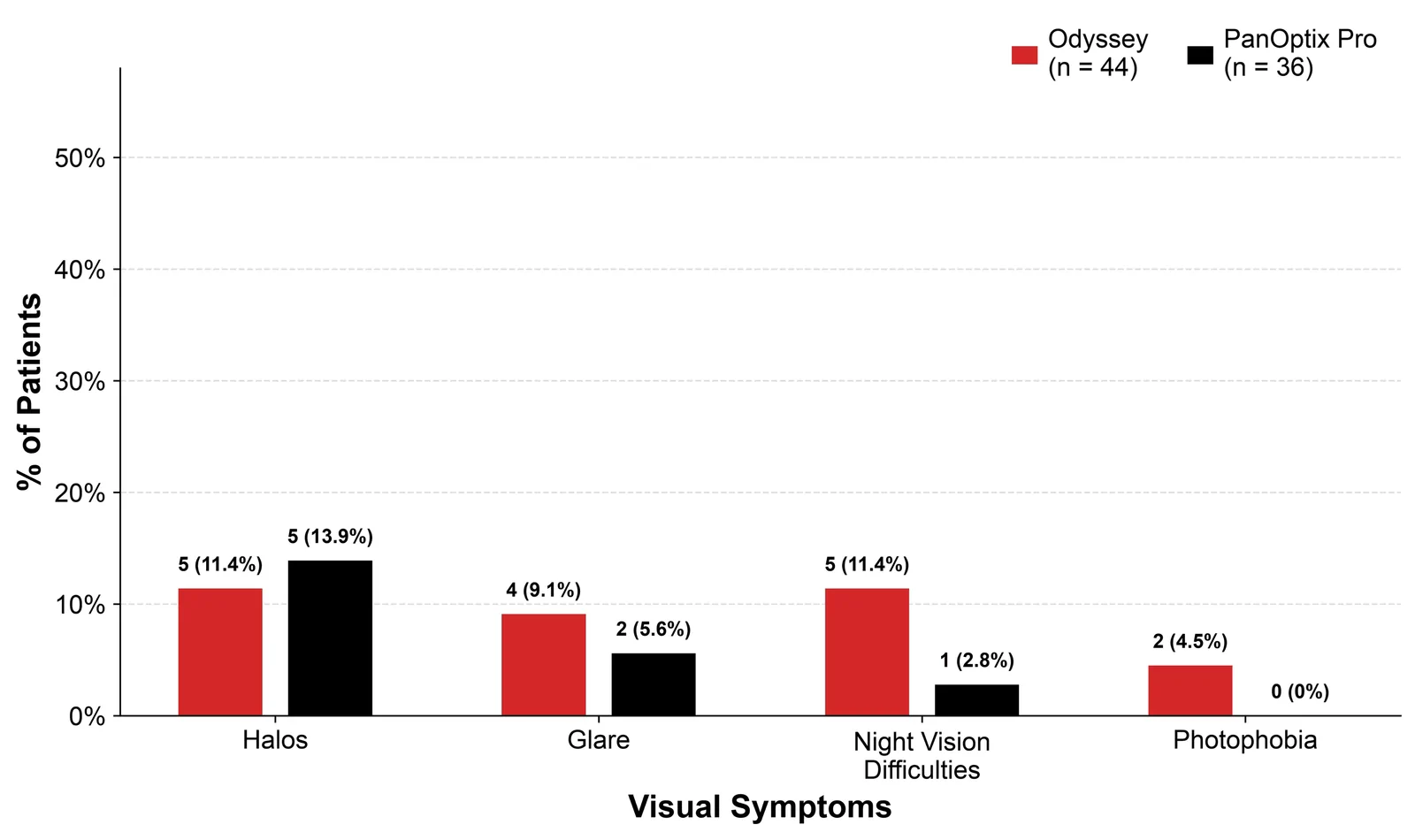
Percentage of patients reporting various visual symptoms at one month postoperatively for the Odyssey (TECNIS Odyssey) and PanOptix Pro (Clareon PanOptix Pro) IOLs. Frequencies (n) are presented outside the parentheses, and the corresponding percentages (%) are shown within the parentheses. Due to the retrospective nature of the study and its limited one-month evaluation period, complete visual symptom data were not available for all included patients. IOL, intraocular lens

No statistically significant differences were identified for any patient-reported photic symptoms at one month postoperatively (Figure 4). Halos were reported at similar frequencies in the Odyssey and PanOptix Pro groups (11.4% vs. 13.9%, respectively; p = 0.747). Night vision symptoms also did not differ significantly between the groups, although they were reported by 11.4% of patients in the Odyssey group compared with 2.8% in the PanOptix Pro group (p = 0.215). Glare and photophobia were infrequent in both groups, with no significant differences observed.

## Discussion

This study compared the visual and refractive outcomes of the TECNIS Odyssey and Clareon PanOptix Pro IOLs. The lenses demonstrated similar proportions of eyes achieving monocular and binocular 20/20 distance and near visual acuity. Given the recent introduction of the Clareon PanOptix Pro IOL, comparative data remain limited; this study provides an early comparison of the visual and refractive outcomes of the Odyssey and PanOptix Pro IOLs.

In our cohort, the Odyssey IOL demonstrated a statistically significant, although clinically small, improvement in postoperative CDVA (mean logMAR, -0.001 vs. 0.039; p = 0.034), whereas near visual acuity outcomes were comparable between the IOL groups. Although a greater percentage of PanOptix Pro eyes achieved the J1+ threshold (56.8% vs. 42.0%), this difference was not statistically significant. These findings may be explained by differences in the optical designs of the two lenses. Specifically, the Odyssey IOL is designed to optimize image quality across a continuous range of vision through its unique echelette surface design, whereas the PanOptix Pro utilizes ENLIGHTEN NXT technology. The echelette design of the Odyssey IOL splits light across a step-like surface, creating multiple focal points over a continuous range of vision. This distribution of light extends the depth of focus, particularly enhancing distance and intermediate vision while maintaining functional near vision [12-14]. This concept is consistent with findings from our previous study, which demonstrated that IOL optical design can extend the range of vision while maintaining excellent distance visual acuity in enhanced monofocal lenses [15]. This interpretation is further supported by previous studies demonstrating enhanced distance and intermediate vision with the TECNIS Symfony IOL (Johnson & Johnson Vision), which employs a similar echelette optical design [11,12,16,17]. In contrast, according to the manufacturer, the PanOptix Pro utilizes ENLIGHTEN NXT technology to reduce light scatter through microsteps in the diffractive structure, allowing light to be redistributed among near focal points [16,18]. Thus, although both trifocal IOLs provide near, intermediate, and distance vision, the Odyssey design emphasizes intermediate and distance vision, whereas the ENLIGHTEN NXT technology of the PanOptix Pro is intended to enhance near vision. This may explain why more Odyssey eyes achieved better distance vision, whereas more PanOptix Pro eyes achieved better near vision in our cohort. Because this was a retrospective, nonrandomized comparison, these optical design explanations should be regarded as hypotheses for the observed differences.

These findings are consistent with the limited preliminary evidence currently available for the PanOptix Pro IOL [19-21]. Previous studies have similarly reported comparable near visual acuity and refractive accuracy between the two IOLs. One conference abstract also reported significantly better distance visual acuity with the Odyssey IOL than with the PanOptix Pro (0.048 logMAR vs. 0.164 logMAR; p = 0.004) at one month postoperatively, supporting the findings of the present study [20]. Overall, these studies have demonstrated favorable distance and near visual outcomes, suggesting that both IOLs provide excellent postoperative visual performance. Although our outcomes were limited to one month of postoperative follow-up, previous literature has reported stabilization of refractive outcomes within a few weeks after surgery [22], supporting the relevance of our findings.

The favorable visual outcomes observed in the Odyssey IOL group are consistent with previous research. Studies evaluating outcomes at one and three months postoperatively have demonstrated high refractive accuracy and excellent uncorrected and corrected distance, intermediate, and near visual acuity following implantation of the Odyssey IOL [8,23-25]. Previous studies have likewise reported favorable binocular distance and near visual acuity, with a high proportion of eyes achieving the 20/20 vision threshold [23]. In addition to these visual outcomes, previous studies have evaluated the dysphotopsia profile of the Odyssey IOL. Although they reported a higher frequency of halos, they also found variable symptom severity and a relatively low percentage of patients describing their symptoms as bothersome [8,23]. In contrast, our study found a lower incidence of photic symptoms. This difference may be attributable to the method of symptom assessment, as we recorded only the presence or absence of symptoms rather than using a standardized patient-reported outcome questionnaire to assess symptom frequency or severity.

No significant differences were observed in patient-reported photic symptoms between the Odyssey and PanOptix Pro IOL groups at the one-month postoperative visit. This finding is consistent with previous studies reporting a low incidence of bothersome symptoms after implantation of the PanOptix IOLs [26-30] and the Odyssey IOL [8,23,25]. However, because visual symptom data were collected retrospectively based solely on the presence or absence of symptoms rather than with a validated questionnaire, this study cannot establish equivalent dysphotopsia profiles between the two IOLs.

This study has several additional limitations. It was conducted retrospectively with a relatively small sample size, and matching substantially reduced the PanOptix Pro cohort from 118 patients (180 eyes) to 49 patients (65 eyes), which may limit the generalizability of the findings. However, this approach enabled better baseline comparability between the two cohorts. Binocular visual acuity data were also limited, although the findings appeared consistent with the monocular results. In addition, intermediate visual acuity was not assessed, and the limited postoperative follow-up period resulted in incomplete visual acuity data for some included eyes, further limiting the robustness of the findings. Because four surgeons performed the procedures, surgeon-related variability may have influenced the results, although this may also enhance their generalizability. For visual disturbances (glare, halos, and night vision symptoms), data were recorded as the presence or absence of symptoms rather than graded using validated questionnaires assessing symptom frequency and severity. Although this approach simplified data collection, it may have increased the risk of underreporting and ascertainment bias. Furthermore, this study did not assess contrast sensitivity or validated patient-reported outcome measures, limiting our ability to fully characterize the comparative performance of the two IOLs. Given the number of comparisons performed across visual acuity thresholds without adjustment for multiple testing, statistically significant findings should be interpreted with caution. Future studies should include prospective, randomized designs with larger cohorts, longer follow-up, and additional assessments of contrast sensitivity, spectacle independence, patient satisfaction, and intermediate visual acuity. Despite these limitations, this study is among the first to directly compare the visual outcomes of the Clareon PanOptix Pro and TECNIS Odyssey IOLs. It was conducted independently without industry funding, in contrast to approximately 50% of the previously published literature evaluating these lenses.

## Conclusions

The TECNIS Odyssey and Clareon PanOptix Pro demonstrated generally comparable early distance, near, and refractive outcomes in this retrospective matched cohort. A small statistically significant difference in CDVA favored the Odyssey IOL; however, its clinical relevance remains uncertain. Prospective studies with longer follow-up, standardized intermediate vision testing, and validated patient-reported outcome measures are needed.

## Appendices

Appendix A 

**Table 2:** GEE analysis. Results of GEE analyses comparing visual acuity and refraction outcomes between the Odyssey (TECNIS Odyssey) and PanOptix Pro intraocular lenses. For cumulative visual acuity threshold analyses (20/X or better), binary outcomes were analyzed using GEE models with a binomial distribution and logit link function. When one treatment group achieved a 100% success rate at a given threshold, Fisher’s exact test was used in place of the GEE model because complete separation can produce unstable or non-estimable logistic regression coefficients. Continuous outcomes, including mean logMAR visual acuity (UDVA, UNVA, and CDVA) and refraction parameters (sphere, cylinder, and SEQ), were analyzed using Gaussian GEE models with an identity link function and an exchangeable working correlation structure to account for the correlation between fellow eyes. Results are presented as effect sizes using regression coefficients (β) or ORs, as appropriate, together with SEs, 95% CIs, Wald z statistics, p-values, and Fisher’s exact p-values. CDVA, corrected distance visual acuity; GEE, generalized estimating equation; IOL, intraocular lens; SEQ, spherical equivalent; UDVA, uncorrected distance visual acuity; UNVA, uncorrected near visual acuity

Outcome	Threshold	Predictor	N (eyes)	Effect size (β or OR)	SE	95% CI lower	Wald z	p-value	Fisher’s exact p-value
Binomial distribution and logit link function									
Postoperative CDVA	20/20 or better	IOL type	92	0.517	0.468	0.206 to 1.294	-1.41	0.1586	0.2834
Postoperative CDVA	20/25 or better	IOL type	92	0.29	0.852	0.055 to 1.541	-1.453	0.1463	0.1536
Postoperative CDVA	20/32 or better	IOL type	92	0	0.73	0.000 to 0.000	-36.314	0	0.2365
Postoperative CDVA	20/40 or better	IOL type	92	0	1.016	0.000 to 0.000	-24.398	0	0.4891
Postoperative CDVA	20/50 or better	IOL type	92	0	0	0	0	0	1
Postoperative CDVA	20/60 or better	IOL type	92	0	0	0	0	0	1
Postoperative UDVA	20/20 or better	IOL type	111	0.459	0.474	0.182 to 1.162	-1.643	0.1004	0.1162
Postoperative UDVA	20/25 or better	IOL type	111	0.875	0.417	0.386 to 1.982	-0.321	0.7484	0.5647
Postoperative UDVA	20/32 or better	IOL type	111	0.608	0.51	0.224 to 1.650	-0.977	0.3285	0.3331
Postoperative UDVA	20/40 or better	IOL type	111	0.41	0.649	0.115 to 1.462	-1.375	0.1692	0.1513
Postoperative UDVA	20/50 or better	IOL type	111	0	0.482	0.000 to 0.000	-54.971	0	0.0128
Postoperative UDVA	20/60 or better	IOL type	111	0	0.713	0.000 to 0.000	-34.042	0	0.2432
Postoperative UNVA	20/20 or better	IOL type	95	1.904	0.449	0.790 to 4.588	1.435	0.1513	0.2146
Postoperative UNVA	20/25 or better	IOL type	95	1.339	0.586	0.424 to 4.225	0.498	0.6188	0.5882
Postoperative UNVA	20/32 or better	IOL type	95	1.207	0.807	0.248 to 5.870	0.234	0.8153	1
Postoperative UNVA	20/40 or better	IOL type	95	0	0.732	0.000 to 0.000	-37.616	0	0.2164
Postoperative UNVA	20/50 or better	IOL type	95	0	1.017	0.000 to 0.000	-25.359	0	0.4681
Postoperative UNVA	20/60 or better	IOL type	95	0	1.017	0.000 to 0.000	-25.359	0	0.4681
Preoperative CDVA	20/20 or better	IOL type	126	1.449	0.448	0.602 to 3.487	0.829	0.4072	0.4293
Preoperative CDVA	20/25 or better	IOL type	126	0.961	0.382	0.455 to 2.032	-0.103	0.9177	1
Preoperative CDVA	20/32 or better	IOL type	126	0.842	0.456	0.345 to 2.057	-0.378	0.7058	0.8268
Preoperative CDVA	20/40 or better	IOL type	126	0.789	0.532	0.278 to 2.238	-0.446	0.656	0.7904
Preoperative CDVA	20/50 or better	IOL type	126	0.395	0.862	0.073 to 2.138	-1.078	0.2809	0.4401
Preoperative CDVA	20/60 or better	IOL type	126	0.679	0.933	0.109 to 4.232	-0.414	0.6786	1
Preoperative UDVA	20/20 or better	IOL type	126	0	1.007	0.000 to 0.000	-25.312	0	0.4921
Preoperative UDVA	20/25 or better	IOL type	126	0.167	1.118	0.019 to 1.491	-1.603	0.109	0.0598
Preoperative UDVA	20/32 or better	IOL type	126	0.523	0.523	0.188 to 1.459	-1.239	0.2154	0.2193
Preoperative UDVA	20/40 or better	IOL type	126	0.516	0.437	0.219 to 1.214	-1.515	0.1297	0.0785
Preoperative UDVA	20/50 or better	IOL type	126	0.685	0.394	0.317 to 1.483	-0.959	0.3374	0.2085
Preoperative UDVA	20/60 or better	IOL type	126	0.787	0.406	0.355 to 1.742	-0.591	0.5547	0.2843
Preoperative UNVA	20/20 or better	IOL type	122	1.032	0.552	0.349 to 3.046	0.056	0.955	1
Preoperative UNVA	20/25 or better	IOL type	122	0.682	0.483	0.265 to 1.757	-0.792	0.4282	0.4047
Preoperative UNVA	20/32 or better	IOL type	122	0.897	0.418	0.395 to 2.035	-0.26	0.7948	1
Preoperative UNVA	20/40 or better	IOL type	122	0.78	0.405	0.353 to 1.726	-0.612	0.5402	1
Preoperative UNVA	20/50 or better	IOL type	122	1.205	0.414	0.536 to 2.710	0.451	0.6522	0.2785
Preoperative UNVA	20/60 or better	IOL type	122	1.187	0.426	0.515 to 2.734	0.402	0.6879	0.5709
MRSE accuracy	±0.25 D	IOL type	118	0.895	0.408	1.100 to 5.449	4.814	0.0282	0.0362
MRSE accuracy	±0.50 D	IOL type	118	0.401	0.517	0.542 to 4.113	0.603	0.4374	0.6155
MRSE accuracy	±1.00 D	IOL type	119	-1.242	1.134	0.031 to 2.644	1.2	0.2733	0.3775
Gaussian distribution and identity link function									
Postoperative sphere		IOL type	118	-0.036	0.083	-0.198 to 0.126	-0.44	0.66	
Postoperative cylinder		IOL type	103	0.001	0.078	-0.152 to 0.154	0.015	0.9883	
Postoperative SEQ		IOL type	103	-0.034	0.084	-0.199 to 0.132	-0.398	0.753	
Preoperative CDVA		IOL type	126	-0.002	0.033	-0.065 to 0.062	-0.05	0.9598	
Preoperative UDVA		IOL type	126	0.016	0.074	-0.129 to 0.162	0.219	0.8266	
Preoperative UNVA		IOL type	122	0.011	0.057	-0.101 to 0.123	0.192	0.8477	
Postoperative CDVA		IOL type	92	0.04	0.019	0.003 to 0.076	2.123	0.0337	
Postoperative UDVA		IOL type	111	0.026	0.025	-0.022 to 0.075	1.066	0.2865	
Postoperative UNVA		IOL type	95	-0.022	0.026	-0.073 to 0.030	-0.748	0.4092	
